# Direct electrochemical detection of individual collisions between magnetic microbead/silver nanoparticle conjugates and a magnetized ultramicroelectrode†
†Electronic supplementary information (ESI) available. See DOI: 10.1039/c5sc02259b


**DOI:** 10.1039/c5sc02259b

**Published:** 2015-07-29

**Authors:** Jason J. Yoo, Joohoon Kim, Richard M. Crooks

**Affiliations:** a Department of Chemistry , The Center for Nano- and Molecular Science and Technology , The University of Texas at Austin , 105 E. 24th St. Stop A5300 , Austin , TX 78712-1224 , USA . Email: crooks@cm.utexas.edu ; Tel: +1 512-475-8674; b Department of Chemistry , Research Institute for Basic Sciences , Kyung Hee University , Seoul 130-701 , South Korea

## Abstract

Here, we report on the electrochemical detection of individual collisions between a conjugate consisting of silver nanoparticles (AgNPs) linked to conductive magnetic microbeads (cMμBs) *via* DNA hybridization and a magnetized electrode. The important result is that the presence of the magnetic field increases the flux of the conjugate to the electrode surface, and this in turn increases the collision frequency and improves the limit of detection (20 aM). In addition, the magnitude of the charge associated with the collisions is greatly enhanced in the presence of the magnetic field. The integration of DNA into the detection protocol potentially provides a means for using electrochemical collisions for applications in biological and chemical sensing.

## Abstract 2

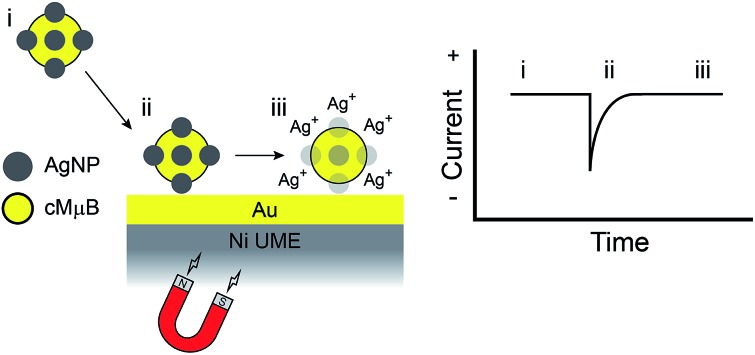
Magnetic fields and silver nanoparticles increase the frequency and current signature of collisions between individual particles and electrode surfaces.

## Introduction

In this paper, we report a new method for amplifying the current signature of collisions between single particles and electrode surfaces.^1^ The specific approach we describe could evolve into a viable means for using single-particle collisions for low-level sensing applications: something that has not yet been achieved. The method involves direct electrochemical detection of silver nanoparticles (AgNPs) conjugated to conductive magnetic microbeads (cMμBs) *via* DNA hybridization. Detection limits as low as 20 aM are achieved due to two factors. First, the presence of multiple AgNPs on each cMμB, and, second, the ability of a magnetic ultramicroelectrode (UME) to increase the rate of mass transport of the cMμBs, relative to diffusion, to the UME surface. These results are significant for the following three reasons. First, they demonstrate the feasibility of direct electrochemical detection of DNA-conjugated AgNPs, which can be used as labels for a variety of electrochemical assays. Second, we provide a detailed analysis of the parameters that control AgNP detection, which are relevant to future bioassays based on collisions. Third, we describe a simple method for preparing magnetic UMEs that will be useful for many different types of applications.

**Scheme 1:**
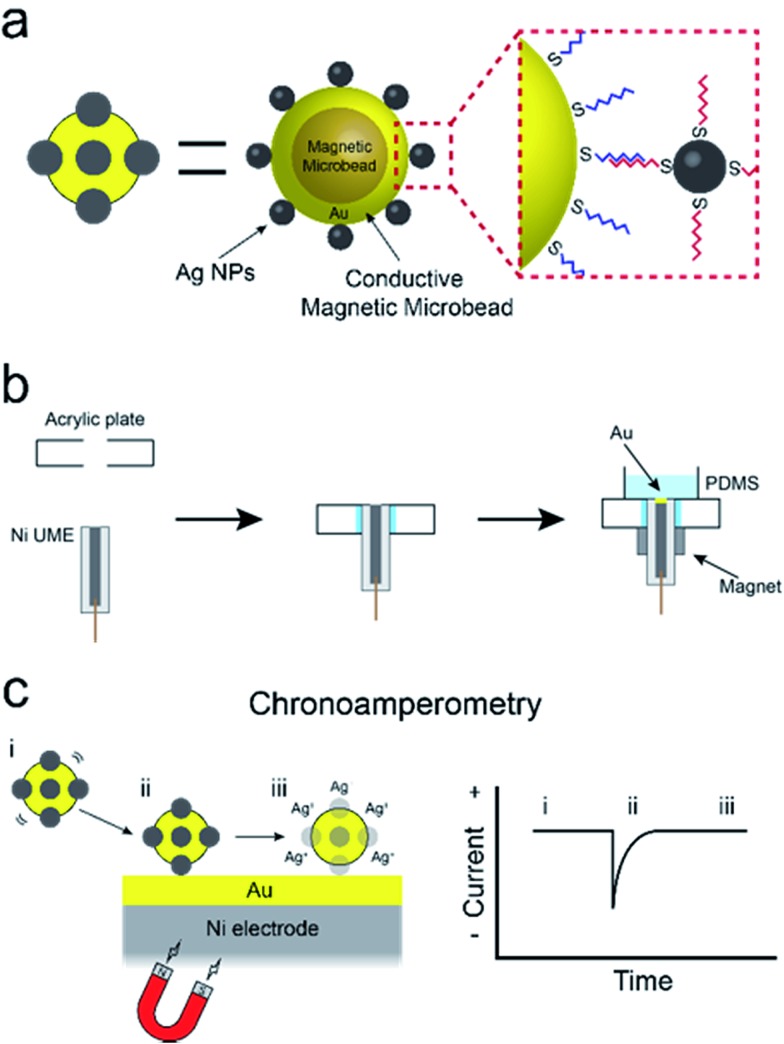


The experiment described in this article is set up as follows. First, as shown in Scheme 1a, the cMμBs are prepared by coating a magnetic microbead with Au to produce a conductive shell, and then this shell surface is modified with AgNPs using DNA hybridization to yield a conjugate of the form: cMμB–DNA–AgNP. Second, a magnetic UME is prepared by coating the surface of a Ni wire with a thin layer of Au, and then placing magnets around the wire to magnetize it (Scheme 1b). Third, as shown in Scheme 1c, an electrochemical cell is configured so that when the cMμB–DNA–AgNP composite is driven to the electrode surface by the magnetic field, the associated AgNPs oxidize more or less simultaneously. This gives rise to an anodic current transient of the type shown in Scheme 1c.

This work was motivated principally by earlier reports from the groups of Lemay,^1^ Compton,^2^ and by one of our own prior studies.^3^ Lemay and co-workers were the first to describe electrochemical detection of collisions between individual nanoparticles and an electrode surface. Specifically, they studied collisions between nonconductive latex beads, having diameters of 150 and 500 nm, and a 5 μm Au UME.^1^ Upon striking the electrode surface, the latex particles were found to irreversibly adsorb to the surface of the UME, and this was signaled by a stepwise decrease in the faradaic current. The current is attenuated because each particle partially blocks the active surface area of the electrode, thereby hindering mass transport of a redox probe (ferrocenemethanol, FcMeOH). This work was extended by Bard and co-workers, who investigated the effect of low electrolyte concentration on collision frequency and amplitude of the current change *via* finite element simulations.^4^

We further extended Lemay's findings by correlating optical tracking and electrochemical measurements of collisions between insulated microbeads and a UME surface.^5^ The collision trajectory was tracked using fluorescence microbeads, and the highest current change was observed when the microbead struck or migrated to the edge of the UME. This finding is consistent with both theory and simulations that predict the highest current flux to be at the edges.^6^ Additionally, Yoo *et al.* showed that it is possible to detect insulated magnetic microbeads (iMμB) in a microelectrochemical device at concentrations as low as 500 zM using a single, moveable magnet placed under the channel of the device.^3^ Pre-enrichment steps collected the microbeads inside the channel inlet and then focused them at the working electrode.

The Compton^2^,^7^–^12^ and Pumera^13^ groups have described a different type of electrochemical collision experiment that is also highly relevant to the findings reported here. In their work, individual or agglomerated metal nanoparticles, usually Ag, strike a UME surface resulting in a burst of anodic current. The important point about this type of approach is that the charge resulting from each collision can be directly correlated to the size of the colliding nanoparticle.

Finally, we note that a number of other research groups have made significant contributions to the study of collisions between particles of various sorts and electrodes. These include the groups of Alpuche-Aviles,^14^ Bard,^15^–^23^ Crooks,^24^–^26^ Koper,^27^ Macpherson,^28^ Stevenson,^29^ Unwin,^30^ and Zhang.^31^

## Results and discussion

### Synthesis and characterization of conductive magnetic microbeads modified with AgNPs (cMμB–DNA–AgNP)

As will be discussed later, it is not possible to carry out experiments like those reported here successfully using iMμBs. This is because the surface of the iMμB is not conductive, and therefore only the AgNPs within ∼1 nm of the electrode would be oxidized. As a result, only a tiny fraction of the total number of AgNPs dispersed on the surface of the iMμB would yield a signal, and assays built on the approach described here would be insufficiently sensitive to be worthwhile.

**Fig. 1:**
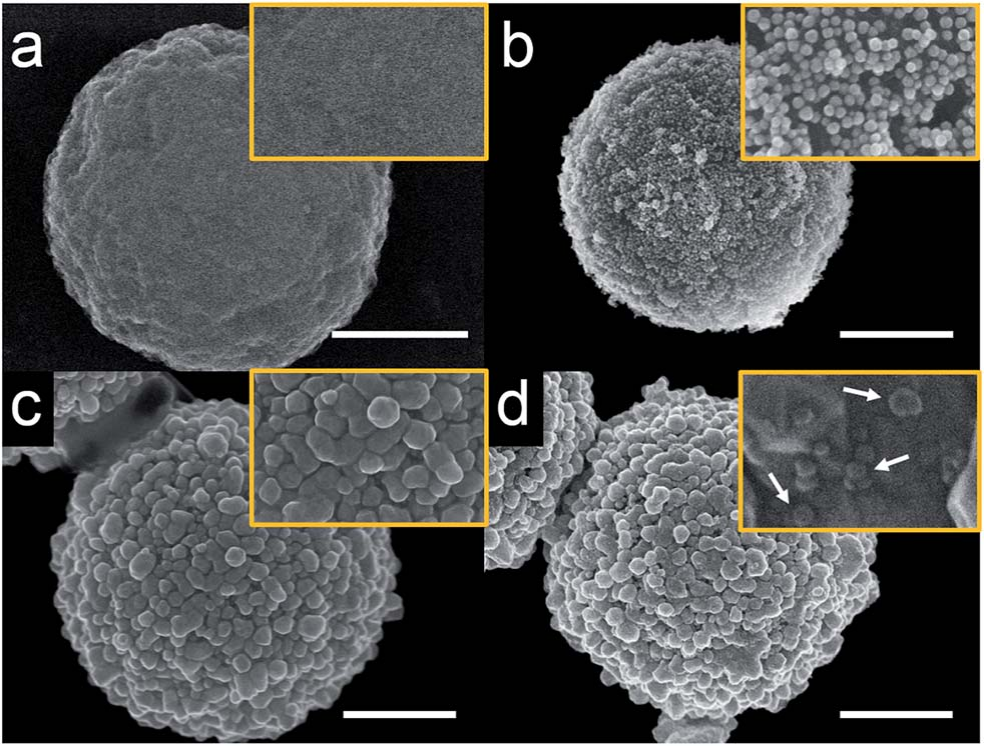
Scanning electron microscopy (SEM) images of MμBs at different stages of synthesis. (a) A carboxylated MμB, (b) after electrostatic adsorption of aminoethanethiol-functionalized AuNPs, (c) after electroless deposition of Au (cMμB), and (d) after functionalization with DNA and AgNPs (cMμB–DNA–AgNP). The white arrows in (d) indicate individual AgNPs. The scale bar is 1.00 μm and the orange box shows an expanded view of each microbead.

The procedure for preparing cMμBs is described in detail in the ESI† but is briefly outlined here.^32^ We start with commercially available iMμBs surface-functionalized with negatively charged carboxylate groups. These are mixed with AuNPs having positively charged 2-aminoethanethiol on their surface (Fig. S1 and S2, ESI†). As shown by the micrographs in Fig. 1a (iMμBs only) and Fig. 1b (iMμBs + AuNPs), this results in electrostatic adsorption of the AuNPs to the surface of the iMμBs. In the second step, the AuNPs act as catalytic sites for electroless deposition of additional Au (Fig. 1c). The average diameter of the MμBs increased from 2.74 ± 0.08 μm to 3.21 ± 0.34 μm after this second step, indicating an Au shell thickness of approximately 235 nm (Fig. S3, ESI†). Finally, the Au-coated MμBs were functionalized with DNA, and then they were mixed with AgNPs having complementary DNA on their surface. As shown by the micrograph in Fig. 1d, this resulted in AgNPs depositing onto the cMμBs to yield the final product: cMμB–DNA–AgNP. Note that concentrations of this conjugate are given in terms of moles of conjugate per liter of solution.

### Direct oxidation of AgNPs *via* anodic stripping voltammetry (ASV)

**Fig. 2:**
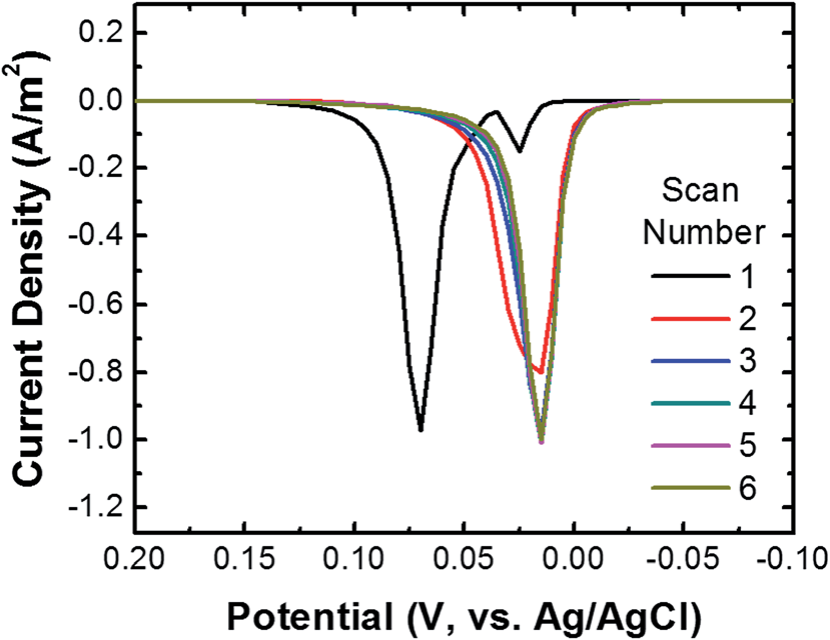
ASVs of cMμB–DNA–AgNP conjugates dropcast onto a GCE. Six consecutive scans are shown. The scans started at –0.20 V and continued to 0.30 V at a scan rate of 50 mV s^–1^ (only part of the scan is shown in the figure). At the conclusion of the individual scans, the electrode potential was stepped back to –0.20 V and held there for 3.0 s before the next scan was initiated. The electrolyte was 100 mM PBCl buffer.

To ensure that most or all of the nanoparticles on the surface of the cMμB–DNA–AgNP conjugate are electrochemically addressable, we carried out the following experiment. The cMμB–DNA–AgNP beads were drop cast onto a glassy carbon electrode (GCE) configured in an electrochemical cell in a face-up orientation. Fig. 2 shows six consecutive ASVs obtained using a single electrode in a solution containing 100 mM NaCl and 10 mM phosphate buffer (pH 7, referred to hereafter as 100 mM PBCl). The black trace is the first ASV, and it exhibits two Ag oxidation peaks: a large peak at ∼70 mV and a much smaller peak at ∼25 mV. It is worth noting, however, that the small peak at ∼25 mV is not present on every first scan. The magnitude of the total charge under these two peaks is 3.37 μC, which corresponds to 6.77 × 10^7^ AgNPs. The shift in peak position for the second and subsequent scans will be discussed later.

To demonstrate the importance of the conductive Au shell in these studies, we carried out a control experiment using iMμBs (no conductive shell) functionalized with AgNPs. These materials were prepared by reacting streptavidin-coated MμB (sMμB, Fig. S4a†) with biotinylated DNA modified AgNPs. An SEM image of the resulting sMμB–DNA–AgNP conjugate is shown in Fig. S4b.† The important result is that when the experiment described in the previous paragraph is carried out using the sMμB–DNA–AgNP conjugate instead of that based on the conductive analog, no detectable ASV current is detected (Fig. S4c†). This confirms the necessity of rendering the iMμBs conductive prior to carrying out collision experiments.

**Fig. 3:**
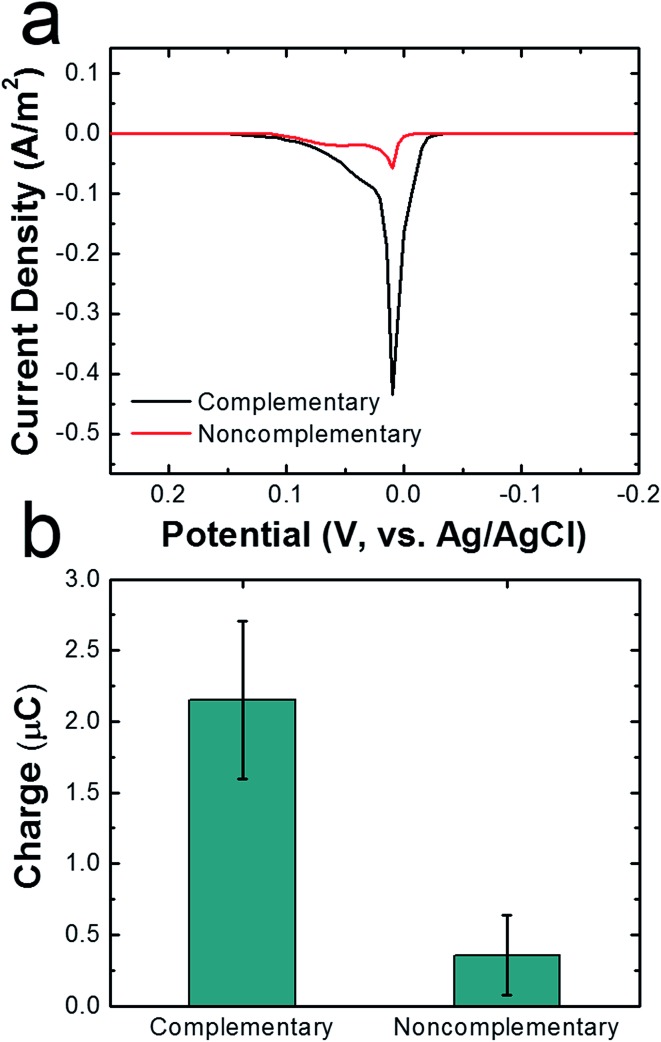
(a) ASVs of cMμB–DNA–AgNP conjugates dropcast onto a GCE. The conjugates were prepared with either complementary (black trace) or noncomplementary (red trace) DNA. The scan rate was 50 mV s^–1^, the initial and final potentials were –0.2 and 0.3 V, and the electrolyte was 100 mM PBCl buffer. (b) Comparison of charges under the ASVs for the conjugates prepared with complementary and noncomplementary DNA. The error bars represent the standard deviation from the mean for three independent trials.

To demonstrate that DNA hybridization is primarily responsible for attachment of cMμBs to AgNPs in the cMμB–DNA–AgNP conjugate, we carried out a control experiment in which noncomplementary DNA was used for the attachment link. ASVs for conjugates built using noncomplementary and complementary DNA are compared in Fig. 3a, and the integrated charge for the two resulting peaks is provided in Fig. 3b. The results clearly show that the average charge from the stripping peak is significantly lower when noncomplementary DNA is used. In other words, there is only a very small amount of nonspecifically adsorbed AgNPs on the cMμBs.

We return now to the second through sixth scans in Fig. 2. These result in just a single peak at ∼20 mV, which is the location of the first small peak in the first scan. For the following discussion we will refer to the peak at ∼20 mV as the first peak and the one at ∼70 mV as the second peak. To understand the origin of these two peaks, the following experiments were performed using the procedure described earlier for the first peak. First, when the potential of the GCE was held at 100 mV prior to returning it to the initial potential of –200 mV and then recording the ASV, the second peak did not appear. Second, when shorter DNA was used to link the cMμBs to the AgNPs, the second peak decreased in size and the first peak increased. On the basis of these experiments, we believe that the origin of two peaks in the ASVs is related to the insulating DNA layer and the possibility that both AgCl and Ag^+^ are products of the electrooxidation of Ag. For the purposes of the present work this is not an especially important point, but it does direct us to hold the electrode potential more positive than the second ASV peak for the collision experiments. More information about the two peaks is provided in the ESI (Fig. S5–S7†).

### Fabrication of a magnetic Ni/Au UME

The detection limit of electrochemical collision experiments is limited by the flux of particles to the electrode surface.^4^,^19^,^22^ Normally, this flux is determined by diffusion, and because particles are large in comparison to molecules, their diffusion coefficients, the hence the limits of detection (LODs) of collision experiments, are generally not very low. To achieve lower LODs, diffusion must be supplemented by a second means of mass transfer like electrophoresis,^4^ pressure-driven flow,^24^,^25^ or, as in the present case, a magnetic force.^32^–^37^ Note that attempts to use electrophoresis and pressure-driven flow to lower LODs have not been very successful.

There are a number of ways one might imagine integrating a magnet into an electrochemical cell for collision experiments. For example, a tiny permanent magnet could be located beneath a microfabricated UME, but this would be very difficult to implement. If the magnet was much larger than the UME, then MμBs would be trapped at locations other than the electrode surface. It is possible to fabricate very small electromagnets, but that is also experimentally challenging and in addition the heat resulting from the windings of the magnet introduces a new variable to the experiment.^38^–^40^ To avoid these types of problems, we simply magnetized the UME itself using an external magnet that focuses the magnetic field at the tip of the electrode.

**Scheme 2:**
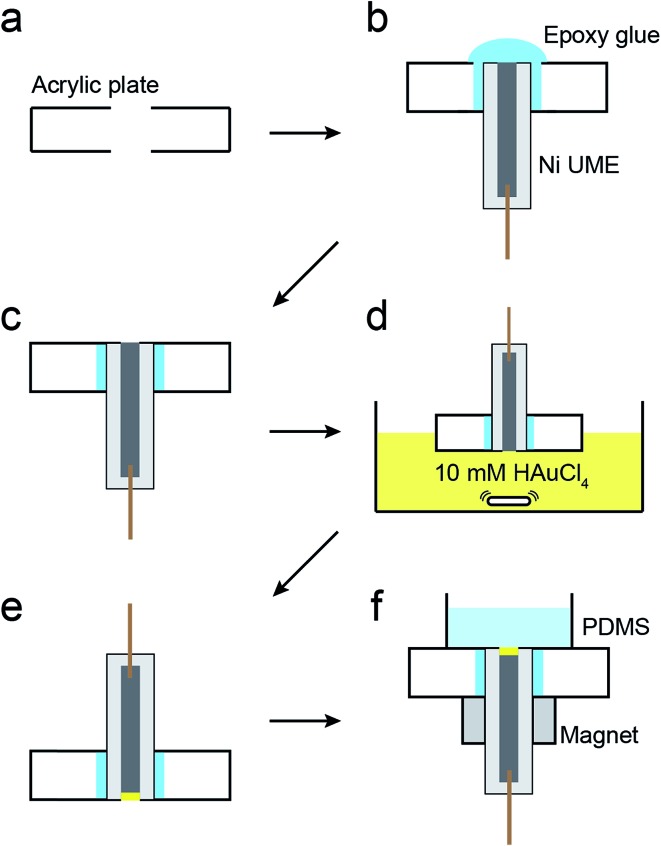


The magnetic UME used here consists of a Ni wire with a thin layer of Au deposited on its tip using galvanic exchange.^41^,^42^ Specifically, a 50 μm Ni UME was prepared by sealing a Ni wire in a glass capillary. As shown in Scheme 2, the Ni UME was then sealed in an acrylic plate using epoxy glue. Next, the surface of the electrode was polished to remove excess epoxy. A thin layer of Au was added by submerging the electrode in a 10 mM HAuCl_4_ solution for 10 s with gentle stirring. This results in spontaneous galvanic exchange between the Ni wire and Au^3+^ in solution. At this point the electrode was washed with a copious amount of DI water and checked under an optical microscope (Fig. S8†) to visually confirm Au deposition by a color change from gray to orange.

**Fig. 4:**
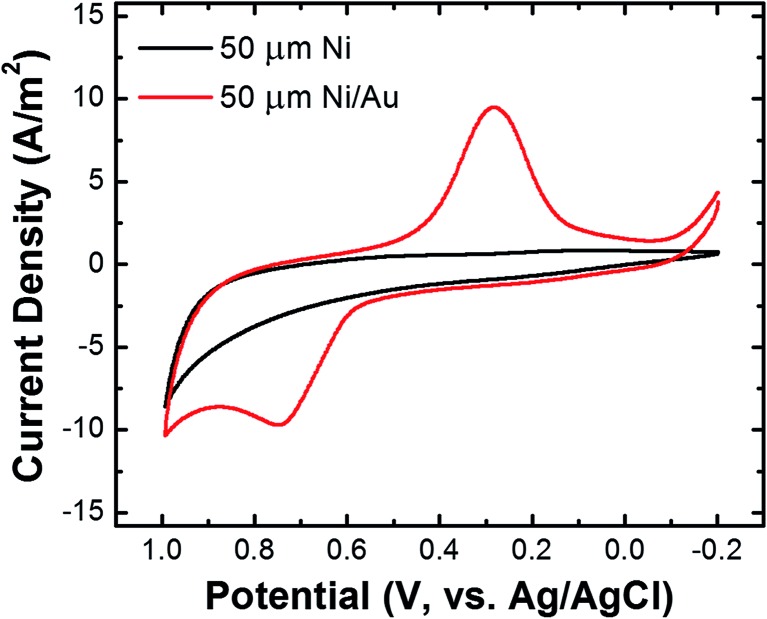
Cyclic voltammograms of a Ni UME before (black) and after (red) electroless deposition of Au. The scans started at –0.20 V, the scan rate was 100 mV s^–1^, and the electrolyte was 100 mM phosphate buffer (pH 7).

The Ni/Au UME resulting from this process was mounted face-up in an electrochemical cell (Scheme 2f). In some cases the electrode was magnetized using ring magnets placed around the UME as shown in the scheme and Fig. S9.† In other cases, the magnets were absent so that control experiments could be carried out. Using the Ni/Au UME (in the absence of the magnets) a cyclic voltammogram (CV) of the Au surface was recorded in 100 mM phosphate buffer solution (pH 7). The red trace in Fig. 4 reveals the characteristic peak potentials (*E*_p_) associated with Au oxidation (*E*_p_ = ∼0.7 V) and oxide reduction (*E*_p_ = ∼0.3 V). This CV can be compared to the black trace in Fig. 4, which shows that these peaks are absent prior to Au galvanic exchange. One final note: of course Ni is less noble than Au, and therefore one would expect peaks associated with Ni oxidation and reduction. Their absence is a consequence of the formation of an oxide of nickel resulting from air exposure.^43^

### Detection of collisions between cMμB–DNA–AgNP conjugates and a magnetic Ni/Au UME

**Fig. 5:**
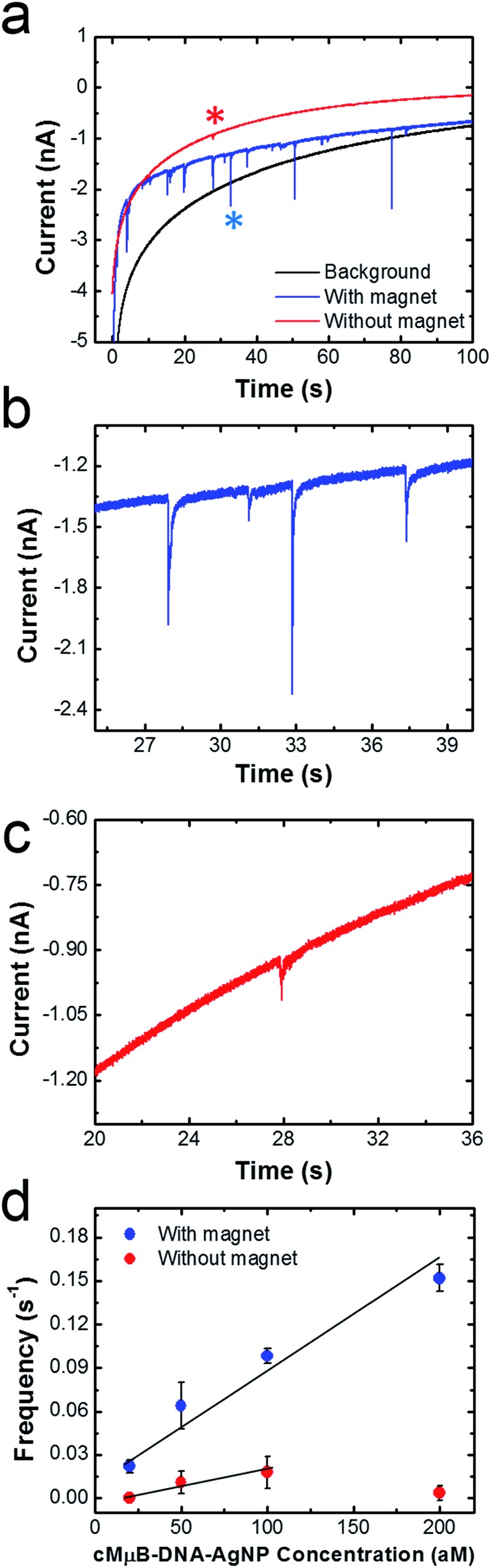
(a) *i*–*t* curves recorded in the absence (black) and presence (red and blue) of 100 aM of the cMμB–DNA–AgNP conjugates. The magnetic field was on for the blue trace and off for the red trace. The potential of the Ni/Au UME was held at 0.2 V. (b) and (c) are expanded views of the blue and red traces in (a). (d) Plots of frequency *vs.* cMμB–DNA–AgNP concentration. The line is the best fit through the indicated data points, and the error bars were determined from three independent collision experiments.

Fig. 5a shows representative chronoamperograms (*i*–*t* curves) for collisions between cMμB–DNA–AgNP conjugates and a Ni/Au UME using a 100 mM PBCl buffer. This experiment was carried out by setting the potential of Ni/Au UME to 200 mV, which is positive of both the first and second ASV peaks shown in Fig. 2. The black trace is a control experiment that was recorded in the absence of the cMμB–DNA–AgNP conjugate, and its shape is similar to that observed in previous collision experiments.^2^ The decrease in the background current as a function of time may be due to blockage of the electrode resulting from progressive accumulation of trace contaminates from solution, or a reduction in activity of the Au UME due to electrodeposition of Ag.^3^ The red and blue traces were recorded in solutions containing 100 aM of the cMμB–DNA–AgNP conjugate plus the PBCl buffer. The blue *i*–*t* trace was obtained in the presence of the magnetic field, and it reveals numerous current transients associated with oxidation of AgNPs. When the magnets are removed from the electrode, the red *i*–*t* trace results. In this case, just a single, small current transient is observed. It is obvious that both the number and size of current transients are much larger when the magnetic field is present.

Fig. 5b and c are expanded views of the *i*–*t* data shown in Fig. 5a in the presence and absence of the magnetic field, respectively. As mentioned earlier, the sharp current transients, which correspond to very fast oxidation of multiple AgNPs per cMμB, are much larger in the presence of the magnetic field (Fig. 5b). Specifically, the average charges for collisions in the presence and absence of the magnetic field are 36.4 ± 33.7 pC and 8.5 ± 6.9 pC, respectively. Although one expects the collision frequency to increase in the presence of the field (*vide infra*), it is not obvious that the magnitudes of the charges should differ so dramatically. We believe there are two possible explanations for this observation. First, the cMμB–DNA–AgNPs may aggregate in the presence of the magnetic field, leading to larger current transients.^44^ Second, it is possible that the cMμB–DNA–AgNPs are in better contact with the electrode or have a longer residence time on its surface in the presence of the field.

The data in Fig. 5d were obtained by carrying out experiments like those described for Fig. 5a, but using concentrations of the cMμB–DNA–AgNP conjugate ranging from 20 to 200 aM. This plot of collision frequency *vs.* the conjugate concentration very clearly demonstrates that the magnetic field enhances the rate of mass transfer of the MμBs to the electrode surface. Although it is difficult to draw a meaningful line through the data points obtained in the absence of the magnet, the ratio of the slopes of the best linear fits through the two sets of data is 4, suggesting that the magnet is responsible for a four-fold increase in signal.

**Fig. 6:**
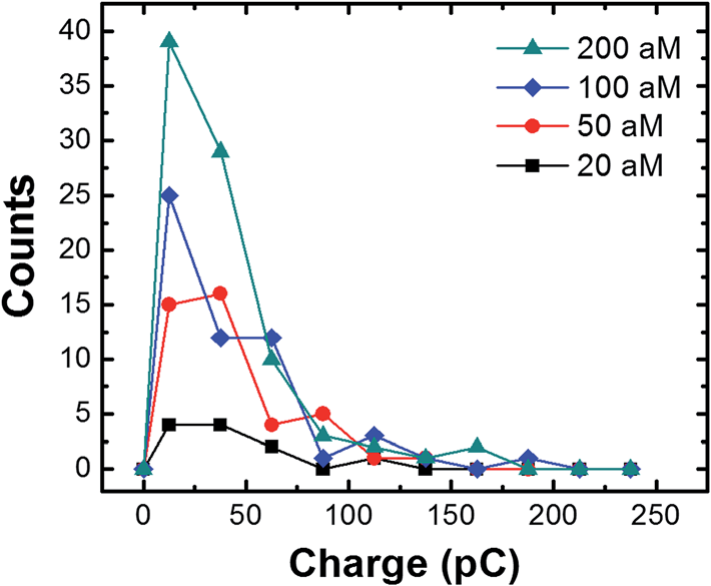
Histograms showing the charges resulting from cMμB–DNA–AgNP collisions (in the presence of the magnetic field) at the indicated concentrations. The average charge at each concentration is 35.4 ± 26.0 pC (20 aM); 37.9 ± 1.3 pC (50 aM); 39.1 ± 37.5 pC (100 aM); and 34.1 ± 32.6 pC (100 aM). The bin size is 25 pC.

The charge resulting from each collision in the presence of the magnetic field was analyzed by measuring the area under the individual current transients as a function of the conjugate concentration (Fig. 6). Regardless of concentration, the majority of the charges range from 20 to 70 pC, with an overall average of 36.4 ± 33.7 pC. The latter value corresponds to oxidation of ∼732 AgNPs.^2^ By measuring the concentration of the AgNPs before and after incubation with the cMμBs (using the NanoSight particle counter), and taking into account the average diameter of the AgNPs (23.3 nm), we find the average number of AgNPs per cMμB to be 3054 ± 260. This value is ∼4 times higher than the average measured from the collision data (∼732 AgNPs). Although the agreement is actually pretty good, the differential is probably due to the different methods used for measurement. The value of 3054 AgNPs/cMμB should include all AgNPs, while the number measured using the collision data only takes into account those that are electrochemically accessible. Electrochemical inaccessibility could arise from AgNPs immobilized on patches of Au that are not in electrical contact with the electrode at the time of collision. Similarly, the presence of the DNA linkers could render some AgNPs too far from the surface of the cMμBs to be oxidized.

**Fig. 7:**
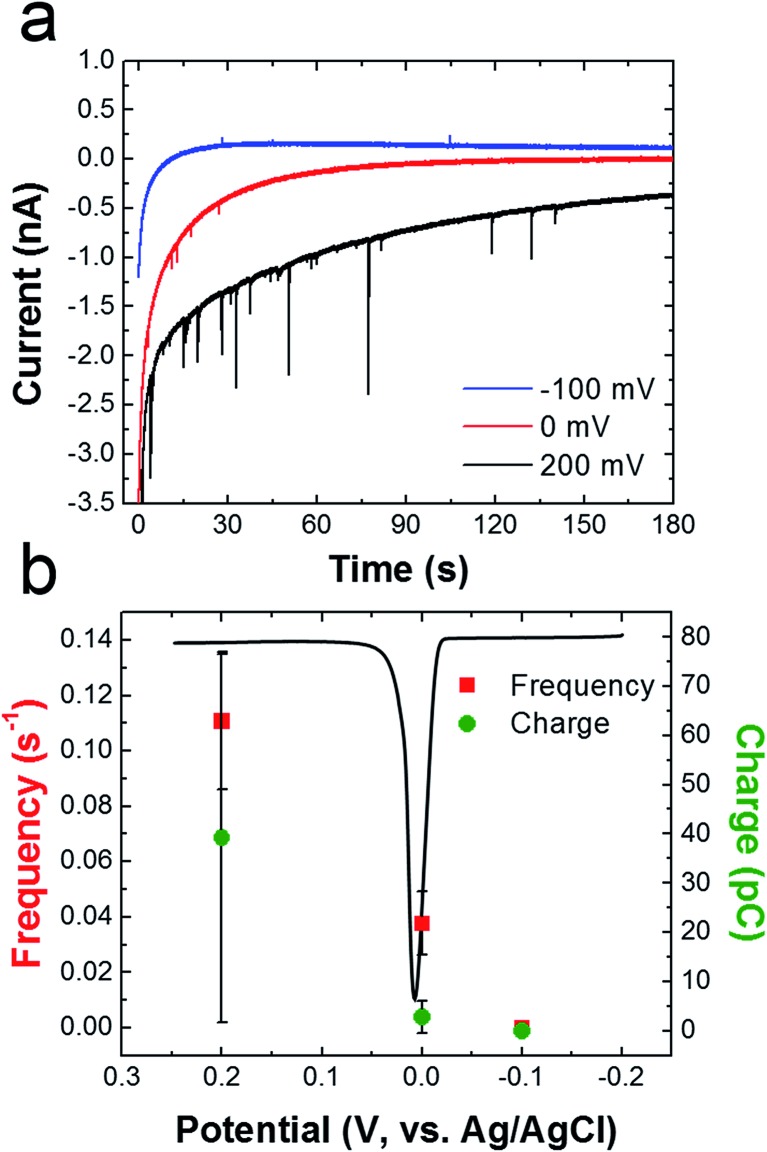
(a) *i*–*t* curves obtained for cMμB–DNA–AgNP conjugate collisions in the presence of a magnetic field. The concentration of cMμB–DNA–AgNP was 100 aM, and the potentials applied to the Ni/Au UME for each experiment are indicated in the legend. (b) Plot of frequency and charge *vs.* the applied potential. The black curve is a representative Ag stripping voltammogram obtained for AgNPs dropcast onto a Au macroelectrode (2 mm). The scan started at –0.2 V and ended at 0.3 V. The scan rate was 50 mV s^–1^ and the electrolyte was 100 mM PBCl buffer. The error bars were determined from three independent experiments at each potential.

To determine if oxidation of the AgNPs is dependent on the potential applied to the Ni/Au UME, collision experiments were performed at three different potentials (Fig. 7). Fig. 7a shows representative *i*–*t* traces for these experiments. The blue, red, and black traces correspond to electrode potentials of –100, 0, and 200 mV, respectively. These three potentials were chosen because –100 mV is more negative than the Ag oxidation potential, 0 mV is at the onset of Ag oxidation, and 200 mV is well into the Ag oxidation potential. The black ASV in Fig. 7b was obtained by drop casting AgNPs onto a Au macroelectrode, and it shows the location of the Ag oxidation peak relative to these three potentials. It reveals a sharp anodic peak at ∼20 mV having an onset potential at ∼0 mV.

Fig. 7b shows that the frequency of anodic current transients is significantly higher at 200 mV compared to –100 and 0 mV (red data points). Additionally, the average charge for Ag oxidation is significantly higher at 200 mV. These results are consistent with previous reports of naked AgNP collision experiments, which showed that the collision frequency and charge increase dramatically after the onset potential for silver oxidation.^2^

## Summary and conclusions

In summary, we have described direct electrochemical detection of AgNPs linked to individual cMμBs. Importantly, the use of a magnetized Ni/Au UME increases the flux of this conjugate to the electrode surface, relative to diffusion, and therefore the collision frequency is higher. Moreover, for reasons we can only speculate on, the magnitude of the collisions is also greater in the presence of the field. This has allowed detection of cMμB–DNA–AgNP conjugates down to a concentration of 20 aM, which corresponds to ∼61 fM AgNPs (recall there are ∼3000 AgNPs/cMμB).

In addition to improving the limit of detection for collision experiments through the use of a magnetic field, the other important aspect of this work is that the AgNP labels are linked to the cMμBs *via* DNA. That opens up the possibility of using collision experiments for DNA detection, which we are currently exploring as a possibility.

## Supplementary Material

Supplementary information
