# Supplementary material for: Direct electrochemical detection of individual collisions between magnetic microbead/silver nanoparticle conjugates and a magnetized ultramicroelectrode
Source: Chem Sci. 2015 Jul 29;6(11):6665–71. doi: 10.1039/c5sc02259b (PMC5506620; doi:10.1039/c5sc02259b)
Supplement: Supplementary file 1 [file SC-006-C5SC02259B-s001.pdf]

Electronic Supplementary Material (ESI) for Chemical Science.
This journal is © The Royal Society of Chemistry 2015

Electronic Supplementary Information

17 July, 2015

Ms. ID: SC-EDG-06-2015-002259

Direct Electrochemical Detection of Individual Collisions between Magnetic Microbead/Silver Nanoparticle Conjugates and a Magnetized Ultramicroelectrode

Jason J. Yoo,[1] Joohoon Kim,[2] and Richard M. Crooks[1],*

[1]Department of Chemistry, Center for Electrochemistry, and the Center for Nano- and Molecular Science and Technology, The University of Texas at Austin, 105 E. 24th St. Stop A5300, Austin, TX USA 78712-1224

[2]Department of Chemistry, Research Institute for Basic Sciences, Kyung Hee University, Seoul, South Korea, 130-701

(20 pages)

Table of Contents

Chemicals and materials	S3
Synthesis of positively charged Au nanoparticles	S4
Synthesis of conductive magnetic microbeads (cMμB)	S4
Modification of AgNPs and cMμB with DNA	S5
Preparation of cMμB-DNA-AgNP and sMμB-DNA-AgNP	S6
Fabrication of the magnetic Ni/Au UME	S6
Electrochemistry	S7
Table S1 (DNA sequences)	S9
Figure S1	S10
Figure S2	S11
Figure S3	S11
Figure S4	S12
Two peaks at different potentials in ASV experiments	S13
Figure S5	S17
Figure S6	S18
Figure S7	S18
Figure S8	S19
Figure S9	S19
References	S20

Chemicals and materials. Sodium phosphate monobasic monohydrate (phosphate, 100%), H_2O_2 (30%), polysorbate 20 (Tween 20, 100%), and NaCl (99%) were purchased from Fisher Scientific (Fair Lawn, NJ) and used as received. $HAuCl_4$, tris(2-carboxyethyl)phosphine (TCEP), citric acid, and 2-aminoethanethiol hydrochloride (98%) were purchased from Acros Organics (Geel, Belgium) and used as received. 4-(2-Hydroxyethyl)piperazine-1-ethanesulfonic acid (HEPES), and $NaBH_4$ (98%) were purchased from Sigma-Aldrich (St. Louis, MO) and used as received. Citrate-stabilized AgNPs (20 nm nominal diameter) were purchased from Ted Pella (Redding, CA). The actual size distribution of the AgNPs as measured by electron microscopy is 23.3 ± 3.3 nm (Figure S1a). The oligonucleotides were purchased from Integrated DNA Technologies (Coralville, IA) and diluted to 100 μM with deionized (DI) water.

The streptavidin-coated (Dynabeads M-270 Streptavidin) and carboxylated (Dynabeads M-270 Carboxylic Acid) insulated magnetic microbeads (iMμBs) were purchased from Life Technologies (Grand Island, NY). The neodymium ring magnets were purchased from K&J Magnetics (Model R422, Pipersville, PA). 50 μm Ni wire (99.99%) was purchased from Alfa Aesar (Ward Hill, MA). Capillary tubes for the UME (O.D.=1.5 mm, I.D.=0.86 mm) were purchased from Sutter Instruments (Novato, CA). The zeta potential and the concentration of AgNPs and AuNPs were measured using dynamic light scattering (Zetasizer Nano ZS) and particle

tracking (NanoSight NS500, Malvern, Westborough, MA), respectively. All incubation steps were performed using a Bioshake iQ at 1500 rpm and 25 °C (BioShake, Jena, Germany). DI water, 18.2 MΩ-cm, was used for all experiments and was obtained from a Millipore filtration system (Millipore, Billerica, MA).

Synthesis of positively charged Au nanoparticles. Positively charged AuNPs were synthesized using a previously reported procedure.[1] Briefly, 500 μL of 213 mM 2-aminoethanethiol solution was added to 50 mL of 1.42 mM $HAuCl_4$. After stirring for 20 min, 12.5 μL of a freshly prepared 10 mM $NaBH_4$ solution was added. The solution was stirred vigorously in the dark for 1 h. Afterwards, the AuNP solution was dialyzed for 4 h and filtered (Millex-SV syringe filter unit 5.0 μm, Millipore). The pH and the zeta potential of the AuNP solution changed from 1.8 and 43.2 ± 8.5 mV to 7.0 and 24.4 ± 12.1 mV after dialysis, respectively (Figure S2). The size of AuNPs was found to be 23.1 ± 3.5 nm by scanning electron microscopy (SEM, Figure S1b).

Synthesis of conductive magnetic microbeads (cMμB). The conductive magnetic microbeads (cMμB) were synthesized using a previously reported procedure with a slight modification.[2] First, 3.0 μL of stock carboxylated iMμBs was added to 1.4 mL of the AuNP solution ((3.0 ± 0.2) × 10^{11} particles/mL) and incubated for 12 h. This is the "AuNP deposition" step wherein the AuNPs (positively charged) attach to the surface of the carboxylated

iMμBs (negatively charged) via electrostatic adsorption. After incubation, the AuNP/MμB conjugates were triply rinsed with 1.0 mL of DI water. To fill voids between neighboring AuNPs, this conjugate was pulled to the side of the vial using a magnet and the solution was exchanged with 1.4 mL of 3.0 mM $HAuCl_4$ containing 0.1% Tween 20. After 1 h of incubation, 14 μL of a 200 mM H_2O_2 solution was added three times every 2.5 h to yield a final H_2O_2 concentration of 6 mM. The H_2O_2 was added sequentially to prevent aggregation of the cMμBs. 2.5 h after the final H_2O_2 addition, the cMμBs were triply rinsed with 1.0 mL of DI water containing 0.1% Tween 20.

Modification of AgNPs and cMμB with DNA. AgNPs and cMμBs were modified with thiolated DNA using previously reported procedures.[3, 4] Briefly, for the AgNPs, 5 μL of 100 μM DNA_{TT} in DI water were added to 400 μL of stock AgNP solution (See Table S1 for information about the DNA). After 5 min of incubation, 25 μL of 100 mM citrate-HCl (pH 3) was added twice with a 5 min incubation time between additions. After 25 min of incubation, 50 μL of 250 mM HEPES buffer (pH 7) was added. The AgNP solution was centrifuged at 16,600 x g for 20 min at 20 $^{\circ}$C. The same procedure was used to modify the AgNPs with biotinylated DNA (DNA_B).

To modify the cMμB with DNA_{TC}, 10 μL of TCEP solution (10 mM TCEP in 5 mM HEPES buffer, pH 7) was added to 10 μL of 100 mM

DNA_{TC} in water and incubated for 1 h in the dark. Afterward, 10 μL of DNA/TCEP solution was added to 1.0 mL of cMμB in DI water. Two rounds of 62.5 μL of citrate-HCl (pH 3) were added after 10 and 20 min. After 25 min of incubation, 125 μL of 250 mM HEPES buffer (pH 7) was added to neutralize the pH. The DNA_{TC}-modified cMμBs (cMμBs-DNA_{TC}) were washed with DI water.

Preparation of cMμB-DNA-AgNP and sMμB-DNA-AgNP. To attach the DNA modified AgNPs (DNA_{TT} and DNA_{B}) to the MμBs (cMμB and sMμB), cMμBs-DNA_{TC} and sMμB were incubated with AgNPs-DNA_{TT} and AgNPs-DNA_{B}, respectively, in 100 mM PBCl buffer for 12 h. The final products were cMμBs-DNA-AgNP and sMμBs-DNA-AgNP, where double-stranded DNA binds the AgNPs to the cMμBs while a single strand of DNA is used to bind AgNPs to sMμBs. The modified composites were triply washed with 100 mM PBCl buffer and stored in the same buffer until needed.

Fabrication of the magnetic Ni/Au UME. Magnetic Ni/Au UMEs were fabricated starting with a homemade 50 μm Ni UME. Using a laser-cutter, a 3 mm hole was cut into a cylindrical acrylic plate (diameter = 20 mm, thickness = 6.35 mm). The Ni UME was secured inside the small hole using epoxy glue. After drying overnight, the surface of the Ni UME and the acrylic plate was polished to expose a disk-shaped Ni surface. To deposit Au on the Ni UME, the UME was submerged in a 10 mM $HAuCl_4$ solution for 10.0 s with gentle stirring. Afterward, the electrode was washed with

copious amounts of DI water. To magnetize the electrode, six neodymium ring magnets were placed around the electrode and held in place with a rubber o-ring (Figure S11).

Electrochemistry. Glassy carbon (GC, dia. 3 mm) and Au (dia. 2 mm) macroelectrodes were polished for 2 min prior to every experiment using 0.3 and 0.05 μm alumina polishing powders, and then they were ultrasonicated in 1:1 DI water:ethanol solutions for 5 min. Anodic stripping voltammetry (ASV) was performed using a bipotentiostat (760b, CH Instruments, Austin, TX). A polytetrafluoroethylene electrochemical (PTFE) cell, the GC and the Au macroelectrodes, a Ag/AgCl reference electrode (details below), and a Pt wire counter electrode (CH Instruments, Austin, TX) were used for the ASV experiments. To record the voltammograms, the GC or Au macroelectrode was inserted into the PTFE cell in a face-up configuration and 2.0 mL of electrolyte solution was added. The scan rate for measuring the voltammograms was 50 mV/s.

For chronoamperometric measurements, *i-t* curves were obtained using a Chem-Clamp voltammeter-amperometer (Dagan Corp., Minneapolis, MN) and a PAR 175 Universal Function Generator (Princeton Applied Research, Oak Ridge, TN). Both the potentiostat and the function generator were connected to a Dell Optiplex 380 computer through a PCI-6251 data acquisition board (National Instruments, Austin, TX). Two-electrode cell

connections from a preamplifier were placed inside a Faraday cage, which was constructed of a copper plate and wire mesh. The *i-t* curves were measured using custom software written in LabView 2010 (National Instruments, Austin, TX). The sampling rate for measuring the *i-t* curves was 500 Hz. All potentials reported in this paper were referenced to a Ag/AgCl “leakless” reference electrode (Dionex, 3.4 M KCl, model 66-EE009 “Leakless”, Bannockburn, IL).

Table S1. Sequences of the DNA used in these experiments.

DNA	Sequence (5'-3')
21-mer Thiolated Target (DNA_{TT})	ThioMC6-D/ACT GCT AGA GAT TTT CCA CAT
21-mer Thiolated Capture (DNA_{TC})	ThioMC6-D/ATG TGG AAA ATC TCT AGC AGT
Noncomplementary TT	ThioMC6-D/CAG ATG CAG TGC CCG ATG AGC
10-mer TT	ThioMC6-D/ACT GCT AGA G
10-mer TC	ThioMC6-D/CTC TAG CAG T
Biotinylated (DNA_B)	Biotin-TCA CAG ATG CGT AAA AAA AAA - C3thiol

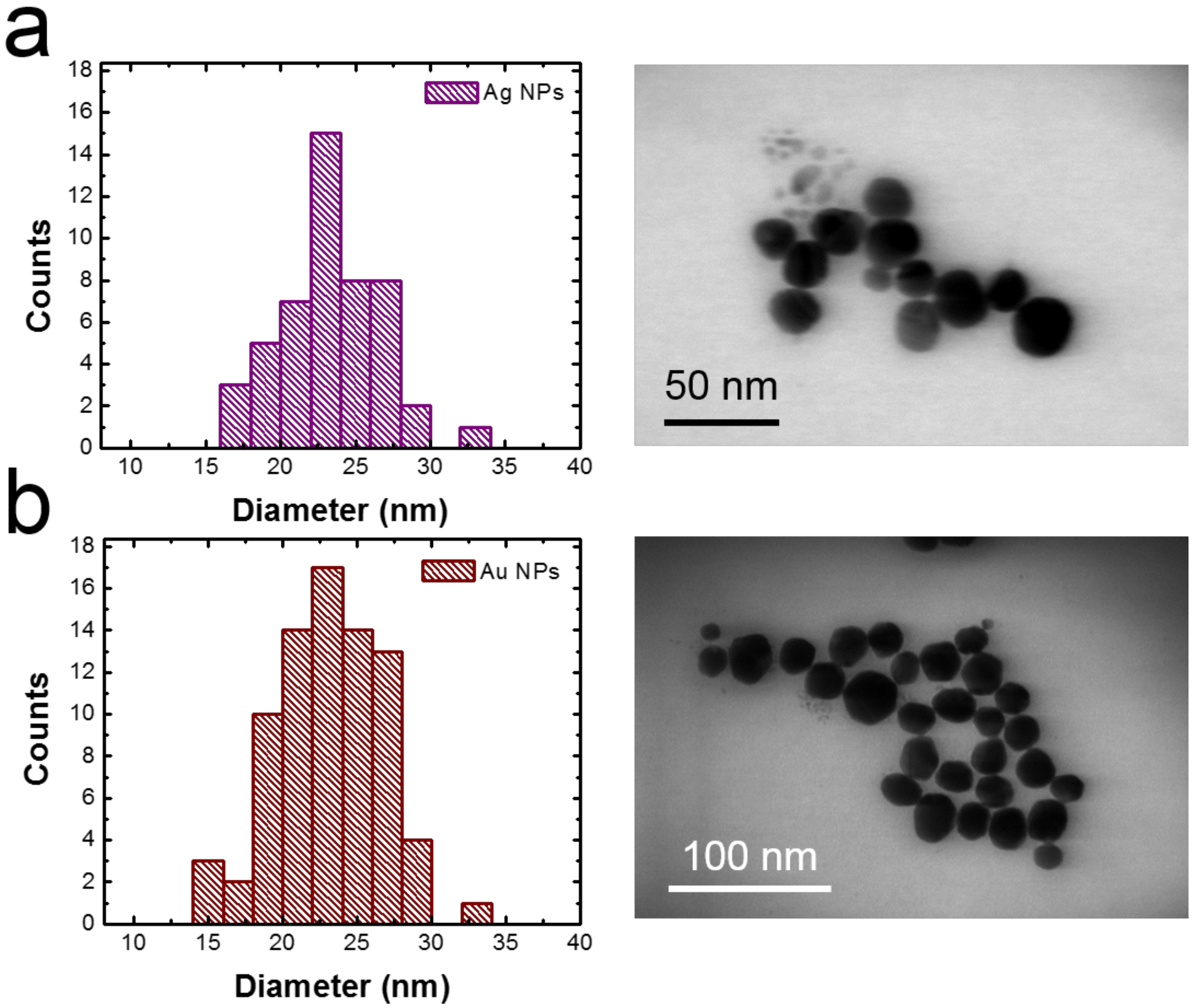

Figure S1. Size-distribution histogram and SEM images of (a) AgNPs and (b) AuNPs. The average diameters are 23.3 ± 3.3 nm and 23.1 ± 3.5 nm for the AgNPs and AuNPs, respectively.

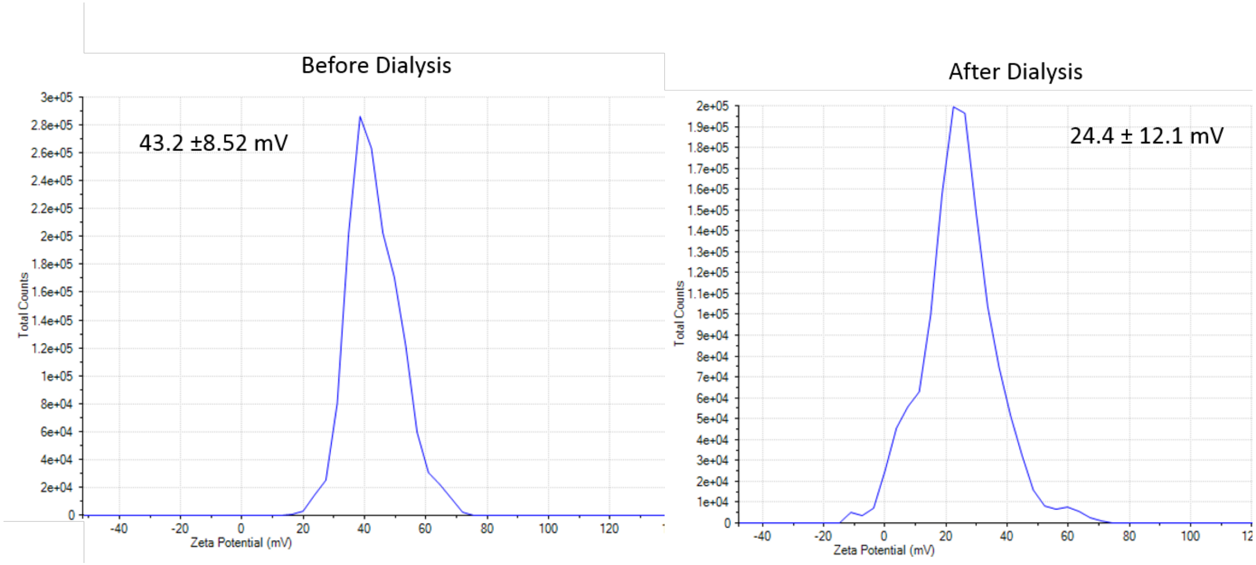

Figure S2. Zeta potential measurements of positively charged AuNPs before and after 4 h of dialysis (against DI water), respectively.

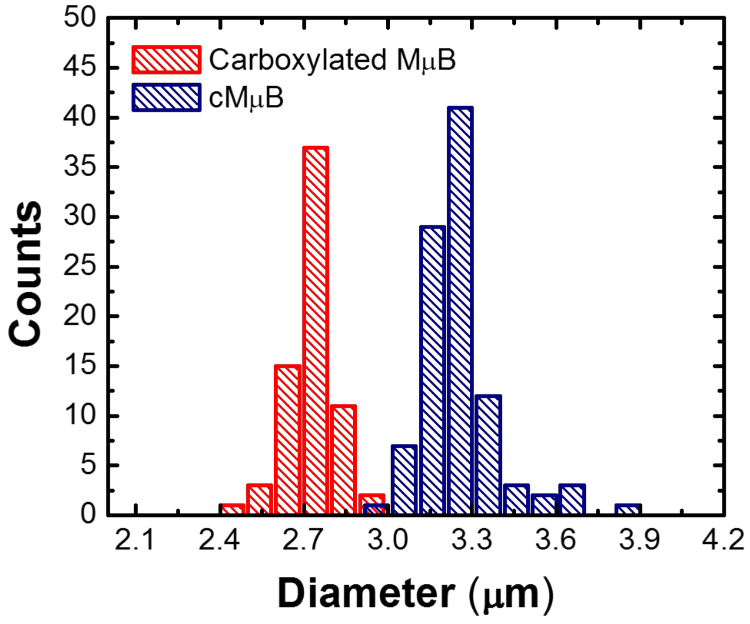

Figure S3. Size-distribution histograms of the carboxylated MμBs (red) and cMμBs (blue). The average diameter increased from 2.74 ± 0.08 μm to 3.21 ± 0.34 μm after deposition of the Au shell. The bin size was 0.1 μm.

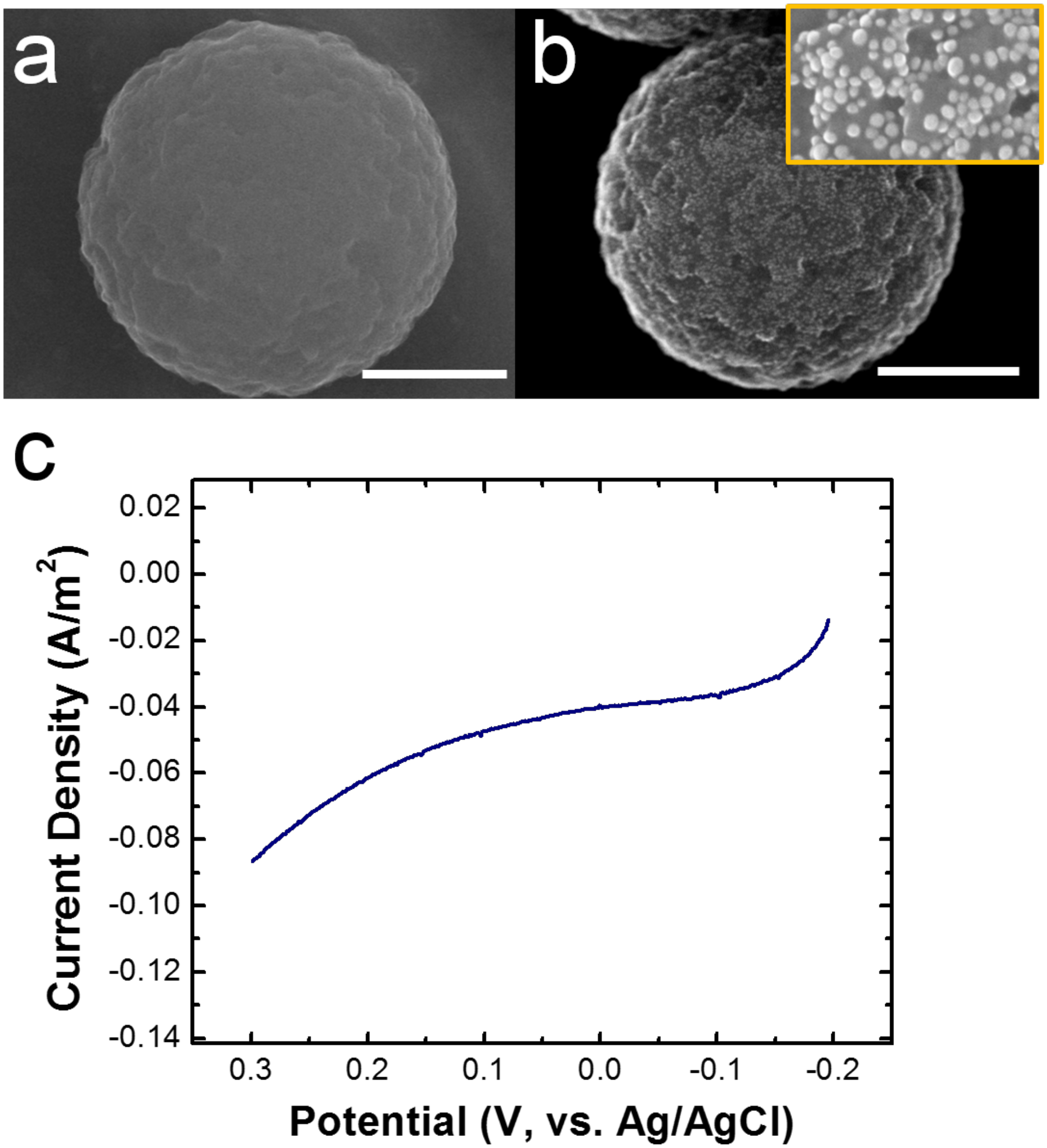

Figure S4. SEM images of streptavidin-coated magnetic microbeads (sMμBs) (a) before and (b) after incubating with AgNPs surface-modified with biotinylated DNA (DNA_B-AgNPs). The scale bar is 1.0 μm. (c) ASV of the sMμB-DNA-AgNP conjugate drop cast on a GC electrode. The voltammogram is plotted without background subtraction. No distinct peaks are observed. The scan rate was 50 mV/s and the electrolyte was 100 mM PBCl buffer.

Two peaks at different potentials in ASV experiments. The appearance of two ASV peaks at different potentials in Figure 2 (main text) was surprising. As a reminder, these ASVs were recorded by dropcasting cMμB-DNA-AgNP conjugates onto the surface of a GC macroelectrode and then recording six consecutive ASVs. In all cases the ASVs started at -0.20 V and proceeded to 0.30 V at 50 mV/s. At the conclusion of each of the individual scans, the electrode was stepped back to the initial potential (-0.20 V) and held there for 3.0 s before initiating the next scan. As shown in Figure 2, the first ASV usually (but not always) results in two peaks, and then subsequent scans yield just a single peak situated at a more negative potential than the dominate peak in the first scan.

During the first scan, the AgNPs, which are coated with a DNA shell, are oxidized to Ag^+ (or perhaps AgCl since the ASVs are carried out in a solution containing 0.10 M Cl^-). After the first scan, the electrode potential is stepped to -0.20 V, which is sufficient to reduce Ag^+ (or perhaps AgCl) back to Ag. Therefore, the second scan oxidizes bulk Ag metal that is in direct contact with the GC electrode surface. That is, the difference between the first and subsequent scans is that during the first scan DNA-coating AgNPs are oxidized, and in later scans bulk Ag not coated with DNA is oxidized. Presumably,

therefore, the presence of DNA or differences in morphology of the Ag lead to the two different ASV peak positions.

To better understand the presence of the two Ag oxidation peaks, we tested the following hypothesis: the local Cl^- concentration (in the vicinity of Ag) is different for the first and subsequent scans. This would be a consequence of the presence of DNA: the negatively charged phosphate backbone of DNA repels Cl^- in its vicinity, and this could lead to a lower local concentration of Cl^- than in subsequent scans when DNA is absent.[5, 6] To test this hypothesis, we carried out three experiments.

In the first experiment, the cMμB-DNA-AgNP conjugate was dropcast onto the surface of the GC macroelectrode, and then six consecutive ASVs were recorded in a solution containing 0.10 M Cl^-. Importantly, however, prior to the first ASV, the electrode potential was initially held at different values for 3.0 s prior to running the ASVs. In all cases, however, the ASVs began at -0.20 V and ended at 0.30 V, and the scan rate was 50 mV/s. Additionally, after each scan, the electrode potential was stepped to -0.20 V and held there for 3 s. As shown in Figure S5, when the potential was held at either -50 or 0 mV, the first scan still produced the second peak. When the electrode potential was held at 50 mV, however, the second peak is greatly diminished and the first peak is larger. When the potential is

held at 100 mV, which is well positive of the second peak, the second peak is absent and only the first peak is apparent. These results suggested that the DNA-modified AgNPs are oxidized at a more positive potential (>50 mV) than naked Ag. This type of behavior has been reported previously, and it is consistent with a lower local concentration of Cl^- in the vicinity of the AgNPs during the first scan due to the presence of DNA.[7-9]

For the second experiment, ASVs were obtained in electrolyte solutions containing different Cl^- concentrations. According to the Nernst equation (eq 1), the Ag oxidation potential will shift toward negative values as the concentration of Cl^- in the vicinity of the Ag increases. Figure S6 shows this effect. Here, naked AgNPs were drop cast onto the surface of a GC electrode, and then ASVs were recorded in solutions containing the indicated Cl^- concentrations. As predicted by the Nernst equation, the ASV peak shifts to more negative values as the concentration of Cl^- increases. This clearly implicates the local Cl^- as important to the genesis of the two ASV peaks.

$$E = E^0 + \frac{RT}{nF} \ln \frac{1}{[Cl^-]} \quad (1)$$

For the third experiment, the length of the DNA linker between the cMμBs and the AgNPs was varied. We reasoned that longer DNA linkers would be more effective at shielding the AgNPs from Cl^-, and that therefore the dominant ASV peak would shift to more negative potentials during the first scan. These

experiments were carried out as follows. First, the cMμB-DNA-AgNP conjugate, wherein the linker DNA was either 10-mer or 21-mer DNA, was dropcast onto the surface of the GC electrode. Second, two consecutive ASVs were obtained in a solution containing 0.10 M Cl^-. The results (Figure S7) show that the shorter DNA linker leads to a smaller second peak. This result seems to confirm the foregoing hypothesis: longer DNA is more effective at shielding the AgNPs from Cl^-.

To summarize, the outcome of the three experiments described above are consistent with our hypothesis that the presence of two peaks at different potentials for the first and subsequent ASV scans in Figure 2 of the main text is a consequence differing local concentrations of Cl^- in the vicinity of the AgNPs. Specifically, DNA is present on the AgNP surface during the first scan. This shields the AgNPs from Cl^- via electrostatic repulsion, and consequently the local concentration of Cl^- is low. Therefore, the relevant redox reaction is given by eq 2. After the first ASV, however, DNA is absent, the local concentration of Cl^- increases, and the relevant redox reaction is given by eq 3. The difference in standard potentials between eq 2 and eq 3 accounts for the two peak positions.

$$Ag^+ + e^- \rightarrow Ag_{(s)} \qquad E^\circ=0.799\ V \qquad (2)$$

$$AgCl_{(s)} + e^- \rightarrow Ag_{(s)} + Cl^- \qquad E^\circ=0.2223\ V \qquad (3)$$

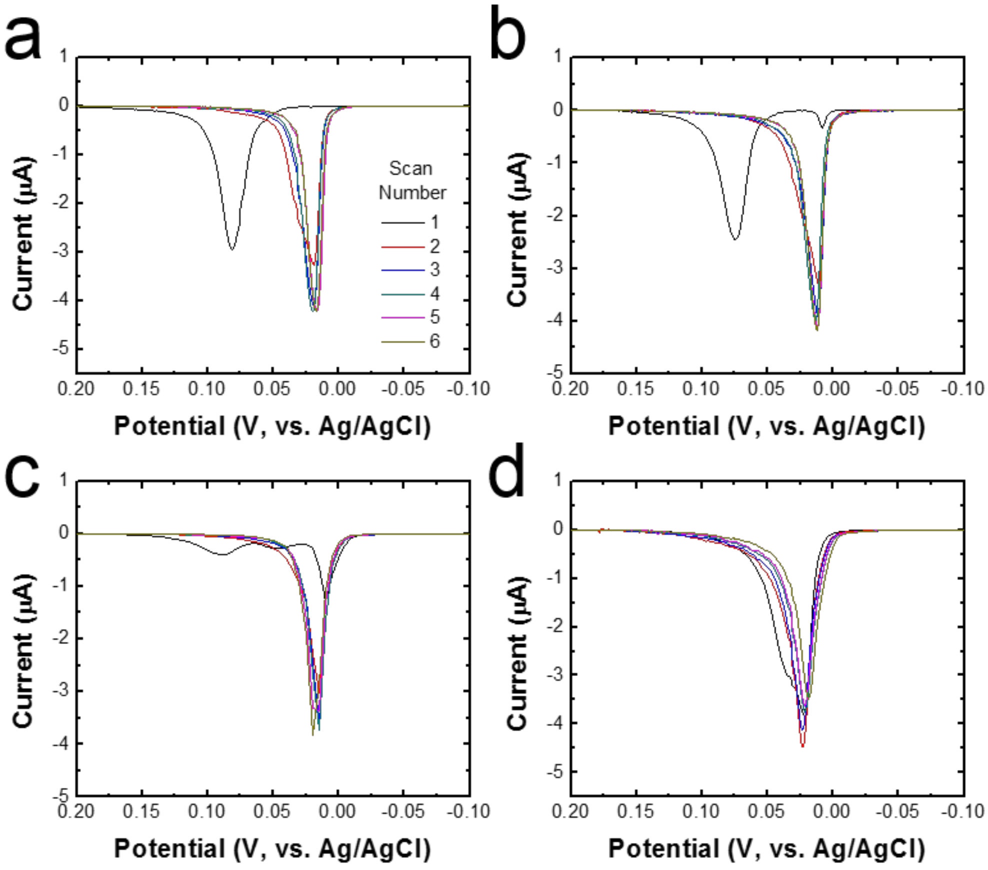

Figure S5. ASVs obtained by dropcasting the cMμB-DNA-AgNP conjugate onto the surface of a GC macroelectrode. Prior to scanning, the electrode potential was held at the following values for 3.0 s: (a) -50 mV, (b) 0 mV, (c) 50 mV, and (d) 100 mV. The experiment was carried out as follows. After holding the potential at one of the values indicated above, the potential was returned to -0.20 V, and held there for 3.0 s, and then scanned to 0.30 V at 50 mV/s. Finally, the potential was stepped back to -0.20 V, held for 3.0 s, and then a second scan was obtained. The remaining scan programs were the same as for the second scan. The electrolyte solution contained 100 mM PBCl.

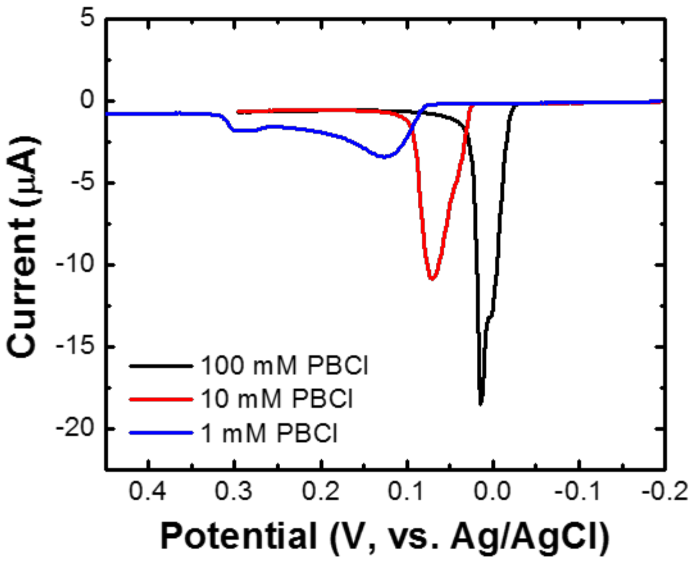

Figure S6. Anodic stripping voltammograms of naked AgNPs dropcast onto Au macroelectrodes in different solutions: 100.0 mM NaCl + 10 mM phosphate (100 mM PBCl buffer, pH 7, black), 10.0 mM NaCl + 10mM phosphate (10 mM PBCl, pH 7, red), 1.0 mM NaCl + 10 mM phosphate (1 mM PBCl, pH 7, blue). The experiment was carried out as follows. AgNPs were dropcast onto the electrode and dried prior to adding 2.0 mL of the solutions indicated above. The potential was held at -0.20 V for 3.0 s and swept to 0.30 V at 50 mV/s. The position of the stripping peak moved more negative with increasing Cl^- concentration.

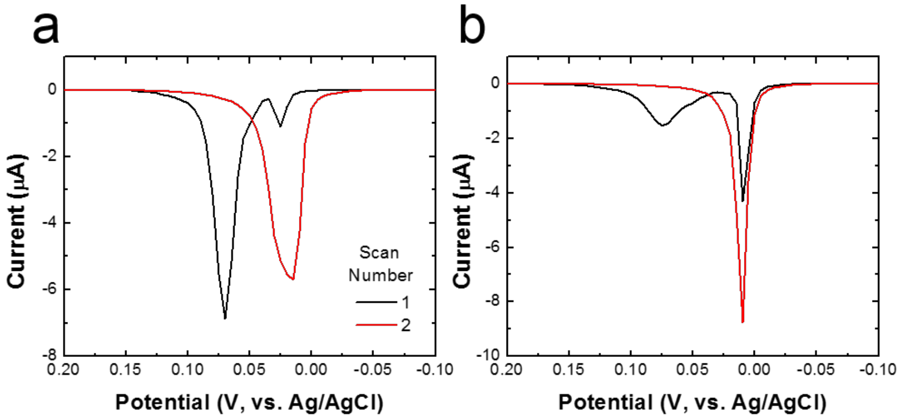

Figure S7. ASVs of cMμB-DNA-AgNP conjugates on GC macroelectrodes. The DNA linker was either a (a) 21-mer or (b) 10-mer. The solution was 100 mM PBCl buffer. The experiment was carried out by dropcasting the cMμB-DNA-AgNP conjugate onto the GC electrode and drying prior to adding the 100 mM PBCl solution. The potential was held at -0.20 V for 3.0 s and swept to 0.30 V at 50 mV/s.

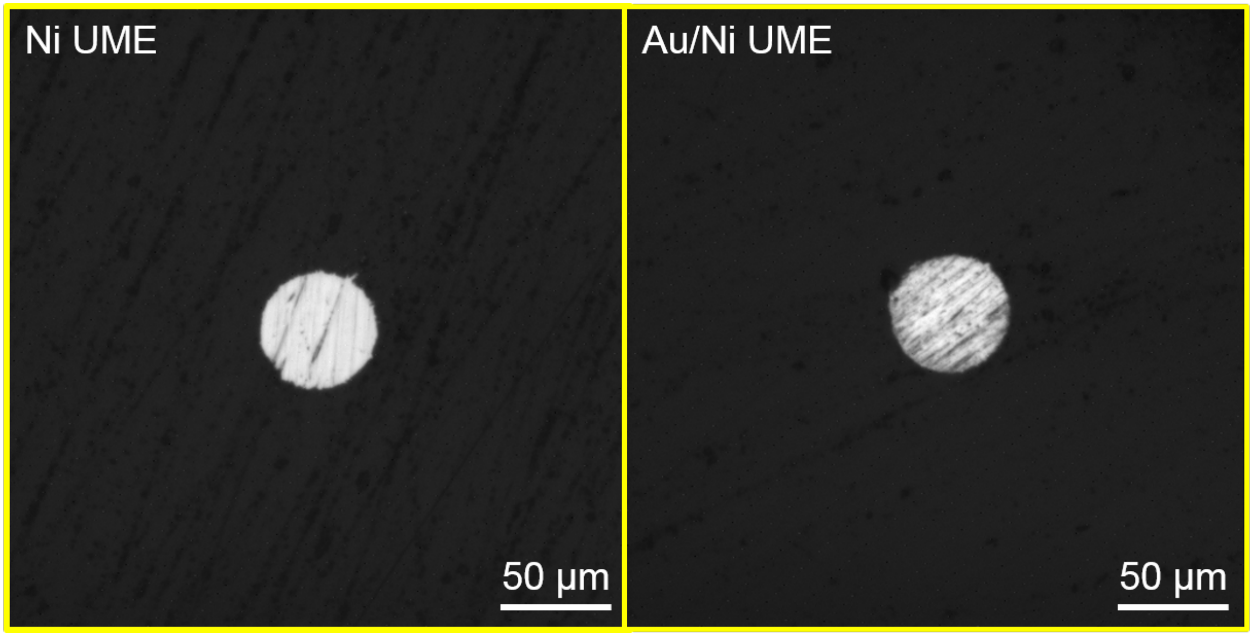

Figure S8. Optical micrographs of Ni and Ni/Au UMEs. The surface of the electrode is rougher after galvanic exchange with Au^{3+}.

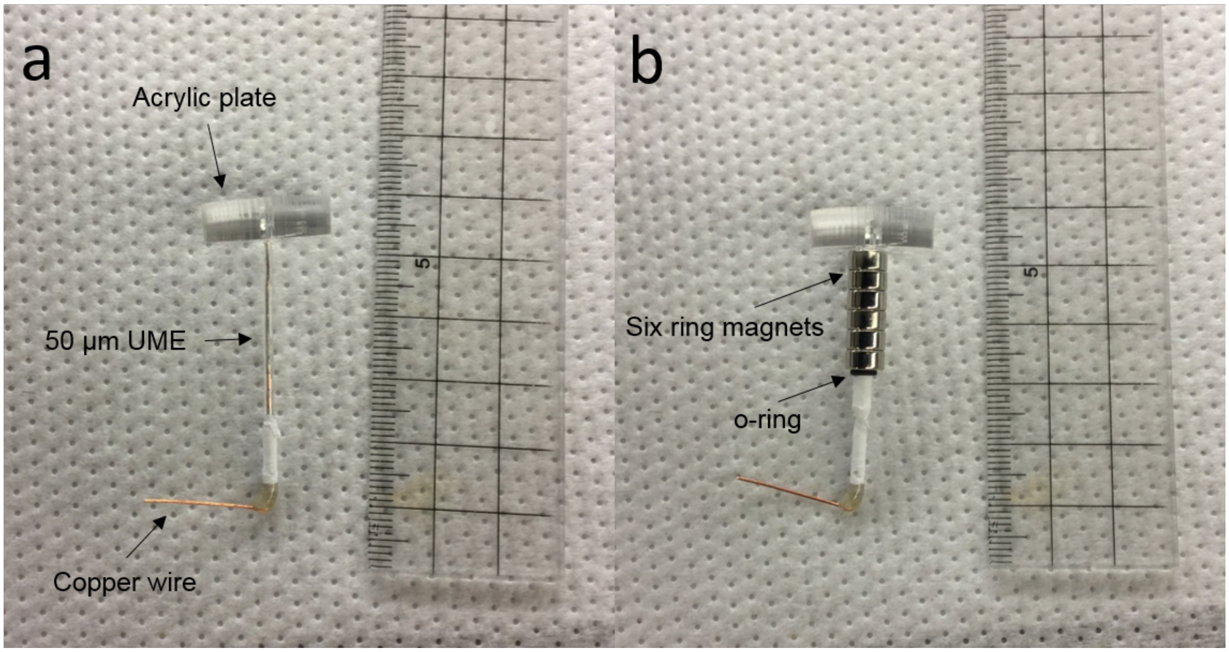

Figure S9. Photographs of the magnetic UME (a) without and (b) with the magnets installed. The o-ring keeps the magnets from slipping off the electrode.

Reference

1. T. Niidome, K. Nakashima, H. Takahashi and Y. Niidome, *Chem. Commun.*, 2004, 1978-1979.
2. H. Shiigi, T. Kinoshita, N. Shibutani, T. Nishino and T. Nagaoka, *Anal. Chem.*, 2014, **86**, 4977-4981.
3. X. Zhang, M. Servos and J. Liu, *J. Am. Chem. Soc*, 2012, **134**, 7266-7269.
4. X. Zhang, T. Gouriye, K. Goeken, M. Servos, R. Gill and J. Liu, *J. Phys. Chem.*, 2013, **117**, 15677-15684.
5. D. S. D. Larsson and D. Spoel, *J. Chem. Theory Comput.*, 2012, **8**, 2474-2483.
6. B. Luan and A. Aksimentiev, *Phys. Rev. E*, 2008, **78**, 021912.
7. D. W. Hatchett, R. H. Uibel, K. J. Stevenson, J. M. Harris and H. S. White, *J. Am. Chem. Soc.*, 1998, **120**, 1062-1069.
8. J. Jin, X. Ouyang, J. Li, J. Jiang, H. Wang, Y. Wang and R. Yang, *Analyst*, 2011, **136**, 3629-3634.
9. H. S. Toh, K. Jurkschat and R. G. Compton, *Chem. Eur. J.*, 2015, **21**, 2998-3004.
